# Unlocking the potential: T1-weighed MRI as a powerful predictor of levodopa response in Parkinson’s disease

**DOI:** 10.1186/s13244-024-01690-z

**Published:** 2024-06-09

**Authors:** Junyi Yan, Xufang Luo, Jiahang Xu, Dongsheng Li, Lili Qiu, Dianyou Li, Peng Cao, Chencheng Zhang

**Affiliations:** 1 Department of Neurosurgery, Clinical Neuroscience Center, Ruijin Hospital, Shanghai Jiao Tong University School of Medicine, Ruijin 2nd Road 197th, 200025 Shanghai, China; 2 Clinical Neuroscience Center, Ruijin Hospital Shanghai Jiaotong University School of Medicine Luwan Brunch, Shanghai, China; 3 Microsoft Research, Unit 4301-4304 AI Tower, No.701 Yunjin Road, 200232 Shanghai, China; 4 Department of Neurosurgery, Center for Functional Neurosurgery, Ruijin Hospital, Shanghai Jiao Tong University School of Medicine, Shanghai, China; 5 Department of Diagnostic Radiology, The University of Hong Kong Hong Kong SAR, Hong Kong, China; 6 Ruijin-miHoYo lab, Clinical Neuroscience Center, Ruijin Hospital, Shanghai Jiao Tong University School of Medicine, Ruijin 2nd Road 197th, 200025 Shanghai, China

**Keywords:** Parkinson’s disease, Magnetic resonance imaging, Levodopa response, Machine learning

## Abstract

**Background:**

The efficacy of levodopa, the most crucial metric for Parkinson’s disease diagnosis and treatment, is traditionally gauged through the levodopa challenge test, which lacks a predictive model. This study aims to probe the predictive power of T1-weighted MRI, the most accessible modality for levodopa response.

**Methods:**

This retrospective study used two datasets: from the Parkinson’s Progression Markers Initiative (219 records) and the external clinical dataset from Ruijin Hospital (217 records). A novel feature extraction method using MedicalNet, a pre-trained deep learning network, along with three previous approaches was applied. Three machine learning models were trained and tested on the PPMI dataset and included clinical features, imaging features, and their union set, using the area under the curve (AUC) as the metric. The most significant brain regions were visualized. The external clinical dataset was further evaluated using trained models. A paired one-tailed *t*-test was performed between the two sets; statistical significance was set at *p* *<* 0.001.

**Results:**

For 46 test set records (mean age, 62 ± 9 years, 28 men), MedicalNet-extracted features demonstrated a consistent improvement in all three machine learning models (SVM 0.83 ± 0.01 versus 0.73 ± 0.01, XgBoost 0.80 ± 0.04 versus 0.74 ± 0.02, MLP 0.80 ± 0.03 versus 0.70 ± 0.07, *p* < 0.001). Both feature sets were validated on the clinical dataset using SVM, where MedicalNet features alone achieved an AUC of 0.64 ± 0.03. Key responsible brain regions were visualized.

**Conclusion:**

The T1-weighed MRI features were more robust and generalizable than the clinical features in prediction; their combination provided the best results. T1-weighed MRI provided insights on specific regions responsible for levodopa response prediction.

**Critical relevance statement:**

This study demonstrated that T1w MRI features extracted by a deep learning model have the potential to predict the levodopa response of PD patients and are more robust than widely used clinical information, which might help in determining treatment strategy.

**Key Points:**

- This study investigated the predictive value of T1w features for levodopa response.
- MedicalNet extractor outperformed all other previously published methods with key region visualization.
- T1w features are more effective than clinical information in levodopa response prediction.

**Graphical Abstract:**

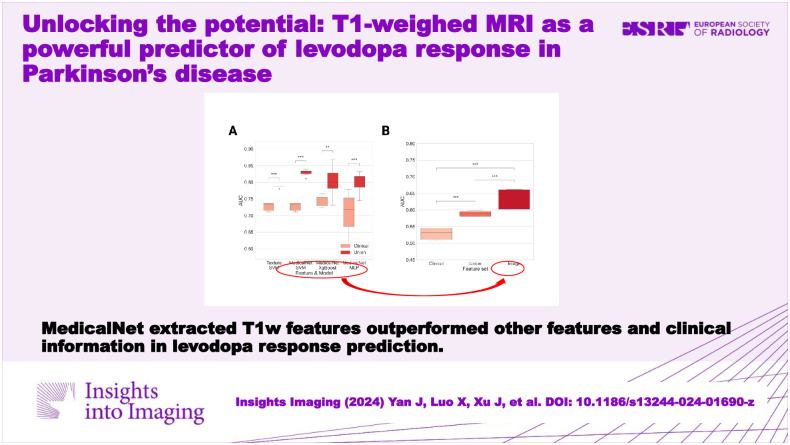

## Introduction

Parkinson’s disease (PD) is a neurodegenerative disorder with a growing prevalence [1]. Its array of symptoms, including tremors, rigidity, bradykinesia, and postural instability, significantly impair patients’ quality of life [1]. Levodopa, a dopamine precursor, is the most used treatment [1–3]. Clinicians often employ the levodopa challenge test (LCT), as its outcomes are crucial for making diagnoses and guiding treatment strategies, particularly that of deep brain stimulation [3]. A predictive model for levodopa response could not only help clinicians determine treatment strategies [4] but also provide insights into potential pathophysiological mechanisms.

T1-weighted MRI is a widely available imaging technique that offers high-resolution brain images. While extensively used in clinical routine for diagnosing and differentiating PD [5–10] and predicting conversion from mild cognitive impairment to dementia [11], its potential for predicting levodopa response has been underexplored. For T1-weighted MRI, Ballarini et al [12] extracted age-corrected gray matter intensity from discriminative voxels between good and poor responders to predict LCT outcomes. Xie et al [13] constructed a morphological brain graph network to fetch individual-level network metrics for LCT result prediction. Furthermore, the PREDISTIM Study Group [4] utilized texture features from 16 subcortical regions of interest (ROIs) to construct feature vectors for each participant to predict LCT results. Although these studies demonstrated the potential of T1-weighted MRI in levodopa response prediction, they either lacked adequate test sets and had limited sample sizes, or did not query the predictive ability of imaging features separately, leaving the underlying potential of T1-weighted MRI in levodopa response prediction unclear. Convolutional neural networks have demonstrated efficacy in brain MRI analysis prediction tasks, including PD diagnosis [14, 15], but have not been utilized in levodopa response prediction. Therefore, the role of T1-weighted MRI in levodopa response should be further evaluated through a more persuasive predictive model.

In this study, we aimed to leverage the Parkinson’s Progression Markers Initiative (PPMI) dataset and an external clinical dataset to evaluate the predictive potential of T1-weighted MRI for levodopa response prediction by comparing classification performance with and without imaging features and identify the underlying brain regions.

## Methods

### Data sources

In this retrospective study, data were sourced in January 2023 from the openly accessible PPMI database (https://www.ppmi-info.org/). PPMI is a multicenter study focused on gathering Parkinson’s progression biomarkers [16]. PPMI participants met specific criteria: PD diagnosis (marked as group ‘PD’ in the PPMI database) and availability of T1-weighted MRI data and MDS-UPDRS III scores for both medication ON and OFF states during the same visit. The exclusion criteria included the lack of a calculable levodopa equivalent daily dose (LEDD) overlapping with the visit time, multiple records for the same patient at one visit, MDS-UPDRS III OFF < 5, and LEDD > 5000. In total, 219 records, with multiple records from the same participants at different visits, were included. An additional dataset of 193 healthy controls from PPMI was included only for age correction.

A threshold of a 30% improvement rate classified the patients into “good” and “bad” responders [13]; the improvement rate was calculated as follows:

$${Improvement}\,{Rate}=	 \frac{{MDS}-{UPDRS}\,{III}\,{OFF}-{MDS}-{UPDRS}\,{III}\,{ON}}{{MDS}-{UPDRS}\,{III}\,{OFF}}\\ 	 \times 100 \%$$

The whole PPMI dataset was randomly split into training and test sets with a ratio of 8:2, ensuring that records from the same participant were in the same set, resulting in 173 and 46 records for the training and test sets, respectively.

**Fig. 1:**
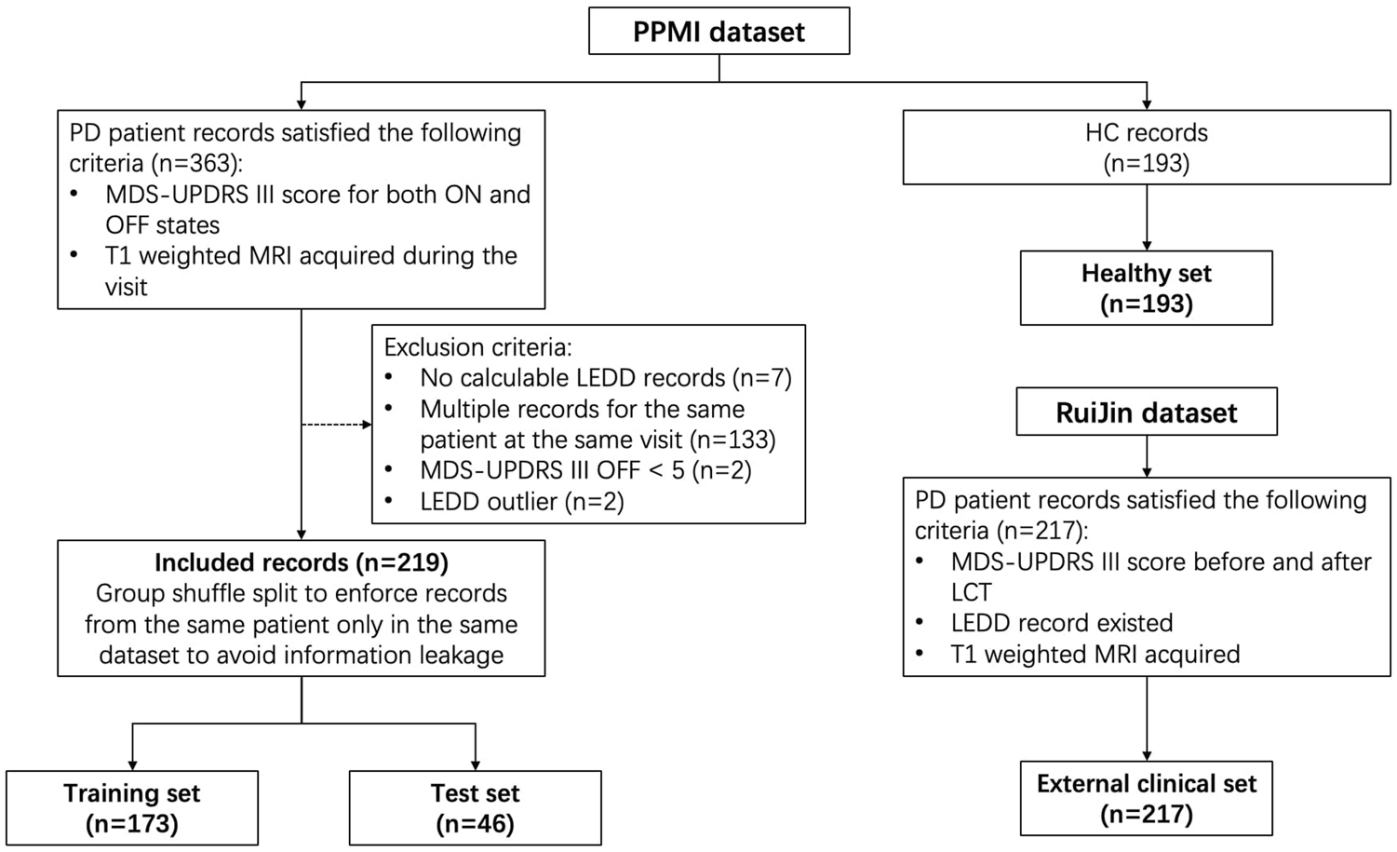
Flowchart of sample inclusion. PD, Parkinson’s disease; HC, healthy control; LEDD, Levodopa equivalent daily dose; MDS-UPDRS III, Movement Disorder Society Unified Parkinson’s Disease Rating Scale Part III

The performance of the output models on actual samples was validated using an external clinical dataset with 217 records from Ruijin Hospital, Shanghai Jiao Tong University School of Medicine, collected between 2017 and 2022. All included participants underwent standard LCT. Notably, these records were collected retrospectively from patients available for deep brain stimulation surgery, which might introduce potential bias to the dataset distribution, with longer disease duration, LEDD, and MDS-UPDRS III scores and a higher proportion of “good” responders (Table 1 and Fig. 1).

**Table 1:** Demographic and clinical information for datasets

	Training Set	Testing set	*p*-value	Clinical set	Healthy set
Source	PPMI	PPMI		Ruijin	PPMI
No. of samples	173	46		217	193
Sex (M/F)	116/57	28/18	0.541	125/92	126/67
Age (years)	64.12 ± 9.22	61.74 ± 9.19	0.123	62.91 ± 9.24	60.29 ± 11.00
Disease duration (m)	41.88 ± 21.61	44.15 ± 23.51	0.537	136.12 ± 56.90	-
LEDD (mg/day)	645.40 ± 426.97	579.82 ± 318.06	0.334	758.04 ± 345.68	-
MDS-UPDRS III OFF	27.51 ± 12.38	25.91 ± 11.67	0.433	56.84 ± 12.06	-
MDS-UPDRS III ON	18.97 ± 10.86	17.43 ± 10.12	0.390	29.82 ± 11.14	-
LCT result (Good/Bad)	86/87	22/24	0.951	201/16	-

*PPMI* Parkinson’s Progression Markers Initiative, *LCT* levodopa challenge test, *LEDD* levodopa equivalent daily dose

T1-weighted MRI scans from PPMI were acquired using 1.5-T (Philips) or 3-T (Siemens) scanners with an isotropic resolution of 1 mm, whereas those from Ruijin Hospital were isotropically acquired using 1.5-T or 3-T scanners (GE) with a resolution of 1 mm to 2 mm.

### Data pre-processing

**Fig. 2:**
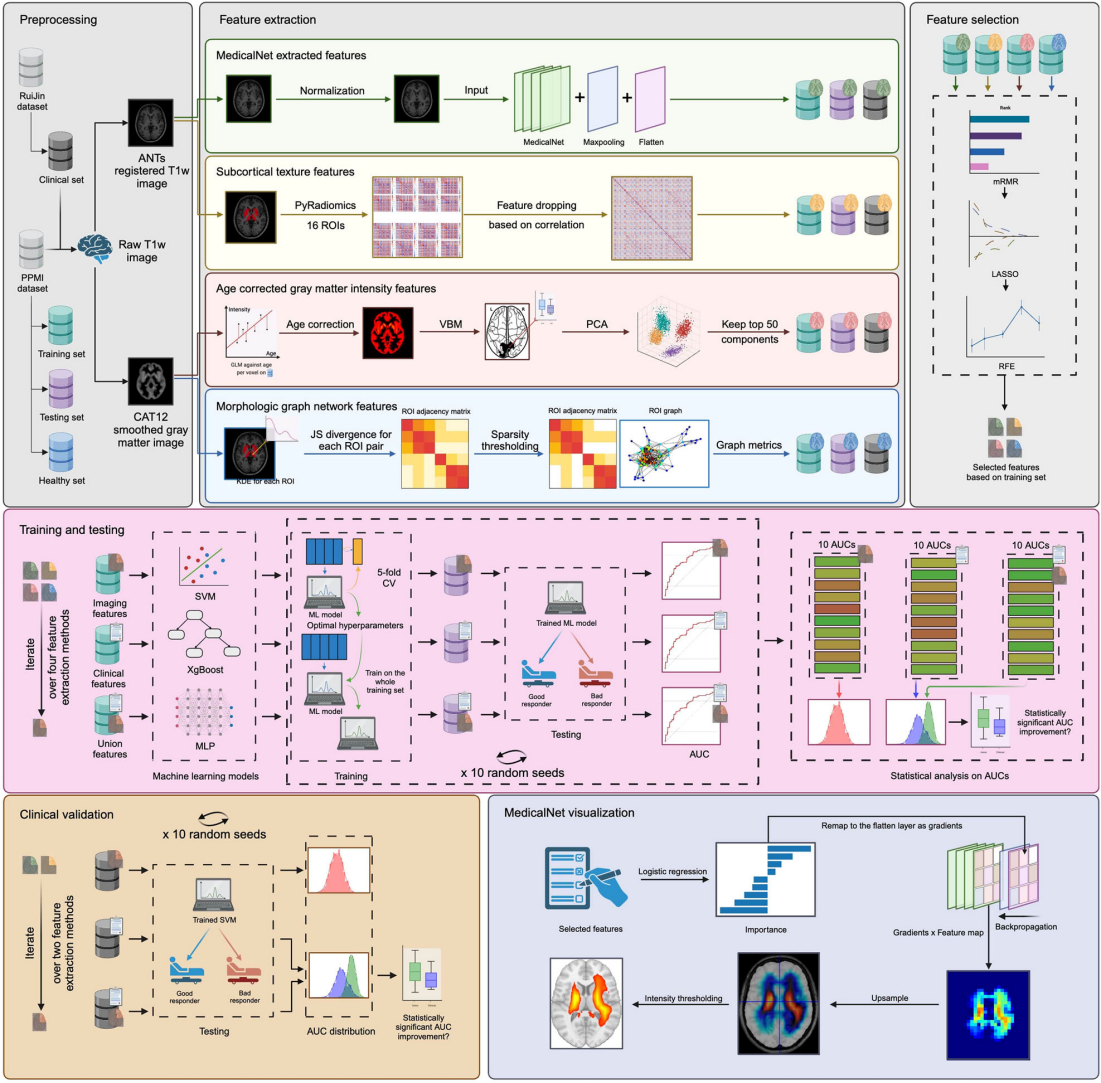
Study design. Two preprocessing methods were performed on T1w images. Four feature extraction methods were then applied to extract features from the preprocessed images. Three feature selection methods were used sequentially to select the most significant features for classification. Three machine learning models were trained on the training set and tested on the test set to predict the category of LCT result (good/bad responder). An external clinical dataset was also included to evaluate the generalizability of the model. The important features of the MedicalNet extractor were visualized. VBM, voxel-based morphometry; CAT12, computational anatomy toolbox; ANTs, advanced normalization tools; ROI, region of interest; PCA, principal component analysis; mRMR, minimum redundancy maximum relevance; LASSO, least absolute shrinkage and selection operator; RFE, recursive feature elimination; SVM, support vector machine; XgBoost, extreme gradient boosting; MLP, multi-layer perceptron

Two image pre-processing pipelines were constructed using Nipype (https://nipype.readthedocs.io/en/latest/) [17] for different feature extraction methods, following previous studies (see Fig. 2). The first one utilized the CAT12 toolbox (http://www.neuro.uni-jena.de/cat/) [18] from SPM12 (https://www.fil.ion.ucl.ac.uk/spm/software/spm12/); the image was segmented into gray matter, white matter, and cerebrospinal fluid, followed by registration to the default template (IXI151_MNI152) in CAT12 at 1.5 mm isotropic voxel size. Spatial smoothing was applied with an 8 mm full width at half maximum Gaussian kernel. The second one utilized ANTs (https://github.com/ANTsX/ANTs) [19, 20]; the image was registered to the PD25 atlas [21–23] using RegistrationSynQuick with an isotropic voxel size of 1 mm.

### Feature extraction

Four feature extraction methods were evaluated, including three from published research and one proposed in this study. Details of the former methods are provided in the Supplementary Materials. In brief, the first one is age-corrected regional gray matter intensity extracted from CAT12 pre-processed images, following Ballarini et al [12], after which principal component analysis (PCA) was used to select the first 50 principal components as features. The second method, proposed by the PREDISTIM Study Group and Chakraborty et al [4, 5] used subcortical ROI textures as PD biomarkers, by extracting and removing highly correlated texture features of 16 subcortical ROIs from ANTs-pre-processed images, encompassing caudate, putamen, thalamus, GPi, GPe, STN, SN, and RN using PyRadiomics (https://pyradiomics.readthedocs.io/en/latest/). The morphological graph was constructed using Kullback–Leibler and Jensen–Shannon divergence following Xie et al [13]. The graph metrics of the individual networks were calculated as features.

To enhance the utility of T1-weighed MRI data, we proposed a feature extraction method based on MedicalNet, a pre-trained ResNet-based deep model tailored for medical images [24]. We replaced the layers originally used for segmentation with a max-pooling layer (kernel size = 8, stride = 8, padding = 0) and a flattening layer. The pre-trained model was fixed and treated as a pure feature extractor. ANTs-pre-processed T1-weighted images (193, 229, 193 dimensions) were input into the model to obtain the output vector as the feature for each sample.

After sequential feature selection, GradCAM [25] was employed to visualize the retained features. The selected features were mapped back to their coordinates as corresponding gradients in the flattening layer. Excluded features were assigned gradients of –0.001. A saliency map was generated and up-sampled for the last convolution layer to visualize the contributing ROIs in the image.

### Feature selection

To refine the feature sets, given their potential redundancy and noise, a feature selection step was necessary for effective classification. Minimum Redundancy - Maximum Relevance (mRMR), least absolute shrinkage and selection operator (LASSO), and recursive feature elimination (RFE) were applied sequentially to the original feature sets. mRMR, based on mutual information, selects features with high relevance to the target and low redundancy [26]. LASSO, based on L1 regularization, compresses unimportant features to zero to achieve feature selection [27]. RFE, based on backward elimination, recursively removes the least important features until the specified number of features is reached.

We sequentially applied these methods to the features extracted from the training set with four feature extraction methods respectively to eliminate irrelevant and redundant features due to the large number of features generated by MRI data, among which LASSO and RFE went through a 5-fold cross-validation to determine optimal hyperparameters. For mRMR, the top 50 features, ranked across the feature sets, were selected for the next step. For LASSO, the optimal regularization parameter *α*∗ was used to fit the model on the entire training set to select the features with non-zero coefficients. For RFE, a logistic regression model representing L2 regularization was used as an estimator in RFE. The entire feature selection process was repeated 10 times to generate a more robust feature set. As a result, the feature number of each extraction method resulted in feature sets being reduced separately.

### Machine-learning models

Machine learning models were trained on a training set using 5-fold cross-validation and tested on a test set to predict the category of the LCT results (good/bad responders). An ablation study was conducted to assess the contribution of T1-weighted MRI data. This involved comparing the classification performance among three feature sets under the same setting: an imaging set, containing features extracted via four methods respectively; a clinical set, encompassing demographic and clinical information including age, sex, disease duration, LEDD, and MDS-UPDRS III OFF; and union set that combined the imaging and clinical sets. All training set features were used to fit MinMaxScaler to scale the training and test set features.

Optimal hyperparameters for each model were determined through 5-fold cross-validation performed on the training set. The specific model was then trained on the entire training set with the optimal hyperparameters and used to predict LCT results for the test set. Repeated experiments were performed to eliminate random effects.

Our study employed three machine learning models—SVM, XgBoost, and MLP—resulting in nine trained and tested models.

### Model performance evaluation

To assess model performance, we used the micro-averaged area under the receiver operating characteristic curve (AUC) as the primary metric. For each feature extraction method and machine learning model, we calculated three AUCs for three test sets generated using three different feature sets. A paired one-tailed *t*-test was performed between the clinical and union sets to evaluate the statistical significance between the clinical and union models.

If any imaging feature set showed a statistically significant contribution (*p* < 0.001), the model was further validated on an external clinical dataset to evaluate its generalizability using the best machine-learning method. More specifically, all models trained in the training stage were fixed without further training and modification, resulting in no additional training in the validation stage. The feature labels to be tested were manually selected according to the feature-selection results of the training set, and feature sets to be validated were built by extracting features from an external set according to feature labels. The external set-generated features were normalized using the MinMaxScaler trained on the training set and inputted into the trained model to predict LCT results.

### Statistical analysis

To evaluate statistical significance between the clinical and union models, a paired one-tailed *t*-test was performed between the two sets, with each containing 10 AUCs generated from 10 random seeds. A *p*-value of < 0.001 was considered statistically significant. All statistical analyses were performed using scikit-learn (https://scikit-learn.org/stable/, version 1.2.1), scipy (https://www.scipy.org/, version 1.10.0), and statannotations (https://github.com/trevismd/statannotations, version 0.5.0) [28].

## Results

### Records inclusion

In this study, we included 219 records from PPMI. The training and test sets encompassed 173 records (mean age, 64 ± 9 years, 116 men) and 46 records (mean age, 62 ± 9 years, 28 men), respectively. The external clinical dataset from Ruijin Hospital included 217 records (mean age, 63 ± 9 years, 125 men), with 201 good and 16 bad responders. The demographic and clinical data of all the datasets are summarized in Table 1.

### Feature extraction

Four distinct feature sets were generated. The age-corrected regional gray matter intensity yielded 50 principal components from discriminative voxels through PCA. Subcortical texture features yielded 86 features from 16 ROIs each, ultimately reduced to 225 features by post-correlation-based feature exclusion. The morphological graph contributed 368 features, whereas the pre-trained model of MedicalNet extracted 13,824 features. There were no differences among repeated selections for all four feature extraction methods.

### Feature selection

The feature extraction steps culminated in four distinct feature sets. The age-adjusted regional gray matter intensity resulted in only one selected feature out of the 50 PCA features. Subcortical texture encompassed two features situated in the right thalamus out of the 225 input features. For the morphological graph, 18 of the 368 features were selected. Of the 13,824 features extracted from MedicalNet, only 9 were selected. Detailed information on the selected features is presented in Table 2.

**Table 2:** Surviving features post-feature selection

Feature name	Description	RFE importance
Gray matter intensity		
PCA_4	Principal component 4 from age-corrected discriminative voxels	7.68e-18
Subcortical texture		
rTHA Gray Level Dependence Matrix LargeDependenceHighGrayLevelEmphasis	Measures the joint distribution of large dependence with higher gray-level values	‒7.80e-5
rTHA Gray Level Size Zone Matrix LargeAreaHighGrayLevelEmphasis	Measures the proportion in the image of the joint distribution of larger-size zones with higher gray-level values	6.96e-5
Morphologic graph		
Nodal Clustering Coefficient		
FAG	Left precentral gyrus	–1.54
PAD	Right postcentral gyrus	–1.08
O2G	Left middle occipital gyrus	0.90
CIPD	Right posterior cingulum	–0.74
F3OG	Left IFG pars orbitalis	1.01
O1D	Right superior occipital gyrus	0.83
T2D	Right middle temporal gyrus	0.71
THAD	Right thalamus	0.65
GAD	Right angular gyrus	0.87
O3G	Left inferior occipital gyrus	–1.05
T1G	Left superior temporal gyrus	0.72
HESCHLD	Right Heschl’s gyrus	0.60
PARA_HIPPOG	Left parahippocampal gyrus	0.84
P1D	Right superior parietal gyrus	–0.64
F1G	Left superior frontal gyrus, dorsolateral	–0.59
F2D	Right middle frontal gyrus	0.88
Degree centrality		
LPCG	Left paracentral lobule	0.73
F3OPG	Left inferior frontal gyrus, opercular part	–0.66
MedicalNet extractor		
ResNet_7020	N/A	–0.38
ResNet_7763	N/A	–1.17
ResNet_3294	N/A	–0.34
ResNet_7509	N/A	1.07
ResNet_7044	N/A	–0.81
ResNet_13074	N/A	0.62
ResNet_874	N/A	0.20
ResNet_12889	N/A	0.35
ResNet_810	N/A	–0.58

### Model performance

**Fig. 3:**
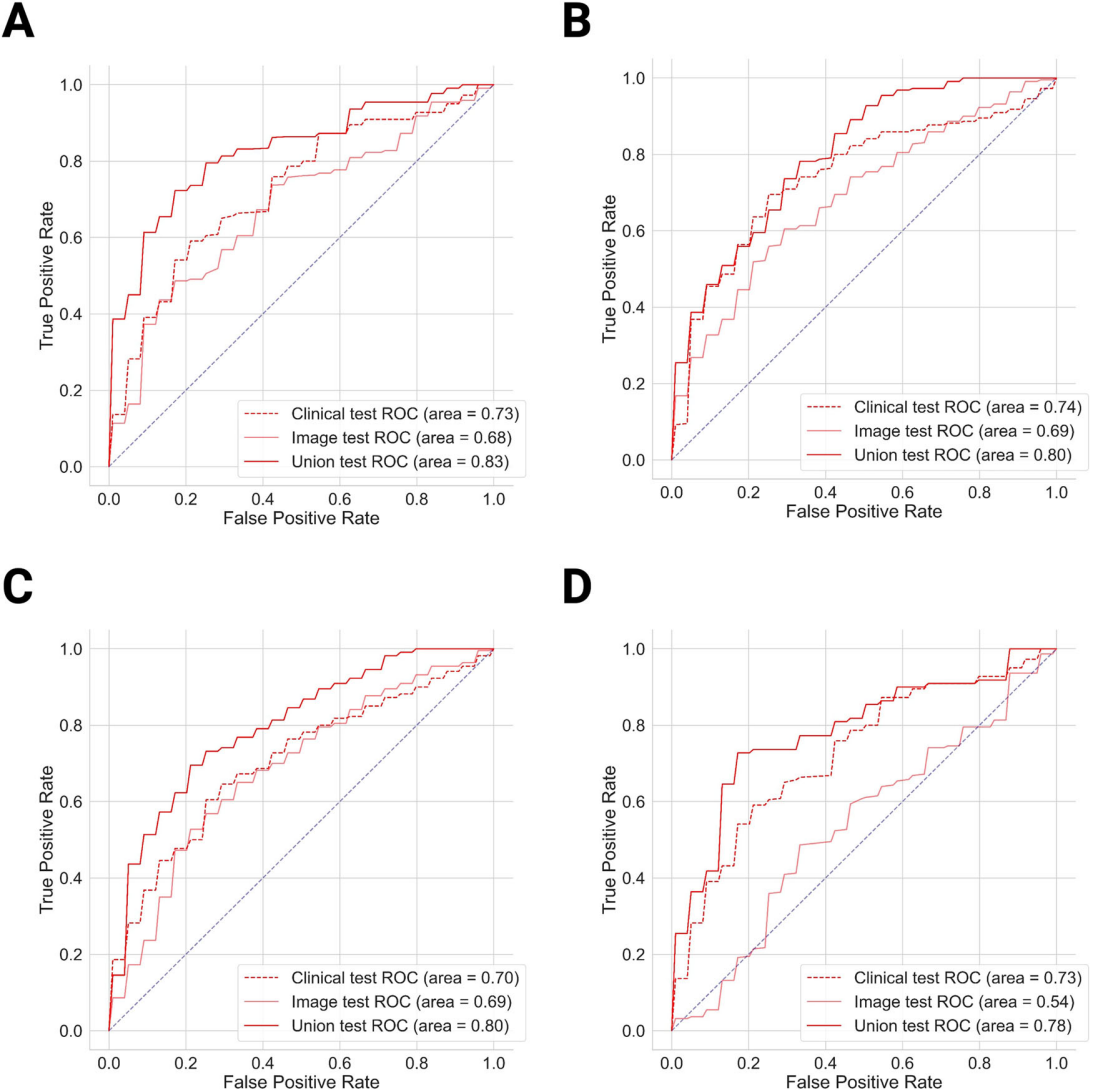
Model performance. **A** ROC curve of MedicalNet feature sets on the test set with SVM. **B** ROC curve of MedicalNet feature sets on the test set with XgBoost. **C** ROC curve of MedicalNet feature sets on the test set with MLP. **D** ROC curve of subcortical texture feature sets on the test set with SVM

Table 3 summarizes model performance on the test set. MedicalNet-extracted features consistently outperformed other feature sets across all three models (SVM Union 0.83 ± 0.01, Clinical 0.73 ± 0.01; XgBoost Union 0.80 ± 0.04, Clinical 0.74 ± 0.02; MLP Union 0.80 ± 0.03, Clinical 0.70 ± 0.07; *p* < 0.001). The best-performing union model, utilizing MedicalNet-extracted features, was SVM, with an AUC of 0.83 ± 0.01 on the test set. For subcortical texture features, only SVM displayed significant improvement (Union 0.79 ± 0.003, Clinical 0.73 ± 0.01, *p* < 0.001). The MLP exhibited a minor but not statistically significant enhancement from 0.70 ± 0.07 to 0.74 ± 0.08. The addition of texture features to XgBoost decreased the AUC from 0.74 ± 0.02 to 0.73 ± 0.03. Neither regional gray matter intensity features nor morphological network features were significantly improved across the three models. The AUC of the improved feature sets are shown in Fig. 3.

**Table 3:** AUCs on the test set

	Imaging	Clinical	Union	*p*-value
Gray matter intensity				
SVM	0.48 ± 0.05	0.73 ± 0.01	0.74 ± 0.002	0.004
XgBoost	0.62 ± 0.04	0.74 ± 0.02	0.75 ± 0.03	0.365
MLP	0.47 ± 0.12	0.70 ± 0.07	0.73 ± 0.03	0.176
Subcortical texture				
SVM	0.54 ± 0.04	0.73 ± 0.01	0.79 ± 0.003	< 0.001
XgBoost	0.64 ± 0.05	0.74 ± 0.02	0.73 ± 0.03	0.899
MLP	0.51 ± 0.09	0.70 ± 0.07	0.74 ± 0.08	0.130
Morphologic graph				
SVM	0.45 ± 0.06	0.73 ± 0.01	0.55 ± 0.03	1.00
XgBoost	0.43 ± 0.03	0.74 ± 0.02	0.55 ± 0.05	1.00
MLP	0.45 ± 0.06	0.70 ± 0.07	0.52 ± 0.05	1.00
MedicalNet extractor				
SVM	0.68 ± 0.03	0.73 ± 0.01	0.83 ± 0.01	< 0.001
XgBoost	0.69 ± 0.03	0.74 ± 0.02	0.80 ± 0.04	< 0.001
MLP	0.69 ± 0.03	0.70 ± 0.07	0.80 ± 0.03	< 0.001

*p*-values were calculated to evaluate the differences between the clinical and corresponding union models

**Fig. 4:**
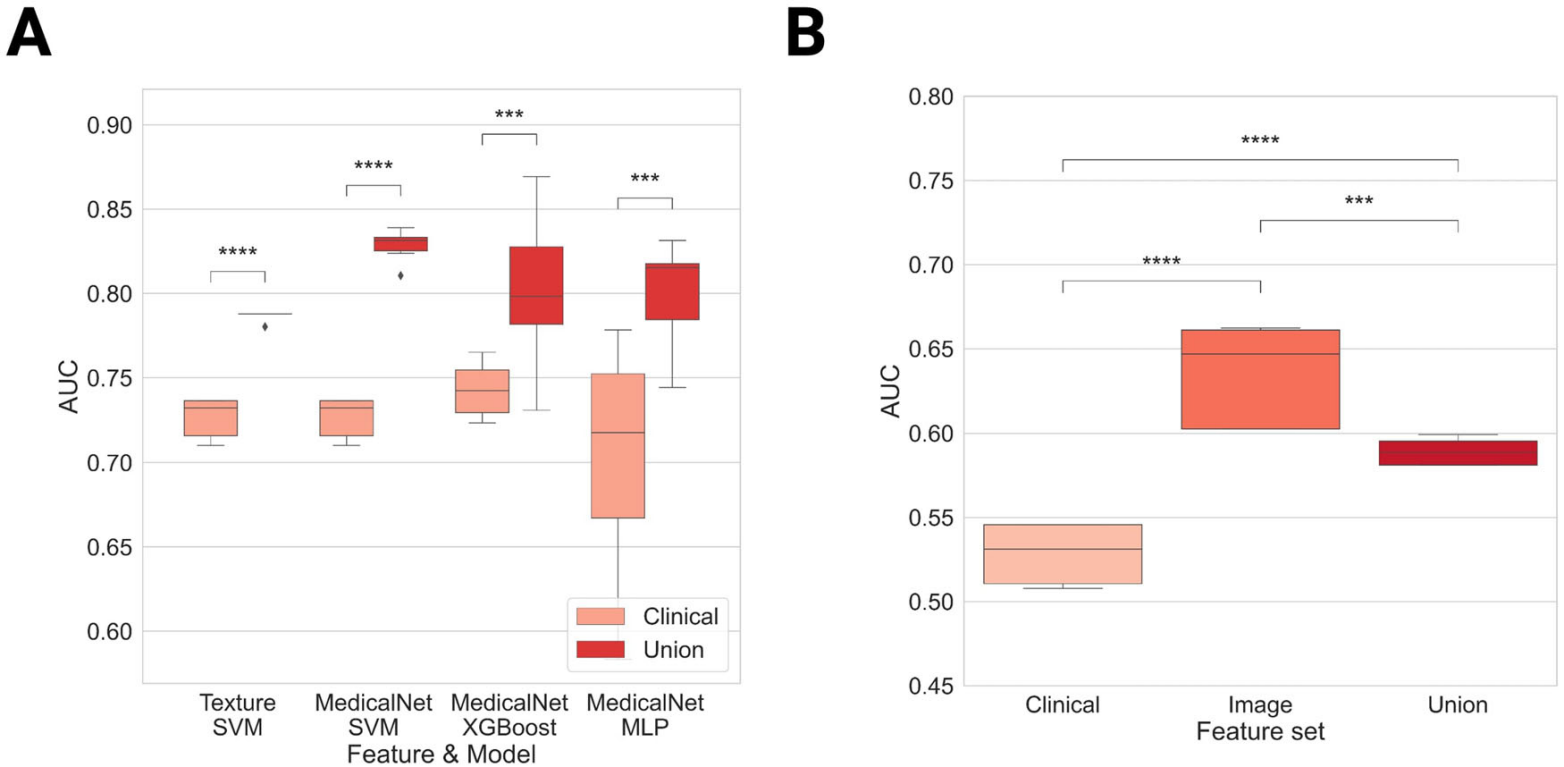
Performance comparison. **A** Box plots of models with significant improvement from the clinical set to the union set on the test set. **B** Box plot of the ROC-AUC distributions of different models using MedicalNet extracted features on the external clinical set. Paired one-tailed *t*-test: ***: 1.00e-04 < *p* < = 1.00e-03, ****: *p* < = 1.00e-04

For the external clinical set (Table 4), both subcortical texture and MedicalNet-extracted features showed a statistically significant improvement with SVM (subcortical texture Union 0.57 ± 0.005, Clinical 0.53 ± 0.01; MedicalNet Union 0.59 ± 0.005, Clinical 0.53 ± 0.01; *p* < 0.001); however, a notable performance drop was observed in both models. The imaging model with MedicalNet-extracted features outperformed all other models (*p* < 0.001), with an AUC of 0.64 ± 0.03 (Fig. 4).

**Table 4:** AUCs on the external clinical set using SVM

	Imaging	Clinical	Union	*p*-value
Subcortical texture				
SVM	0.39 ± 0.08	0.53 ± 0.01	0.57 ± 0.005	< 0.001
MedicalNet extractor				
SVM	0.64 ± 0.03	0.53 ± 0.01	0.59 ± 0.005	< 0.001

### Feature visualization

**Fig. 5:**
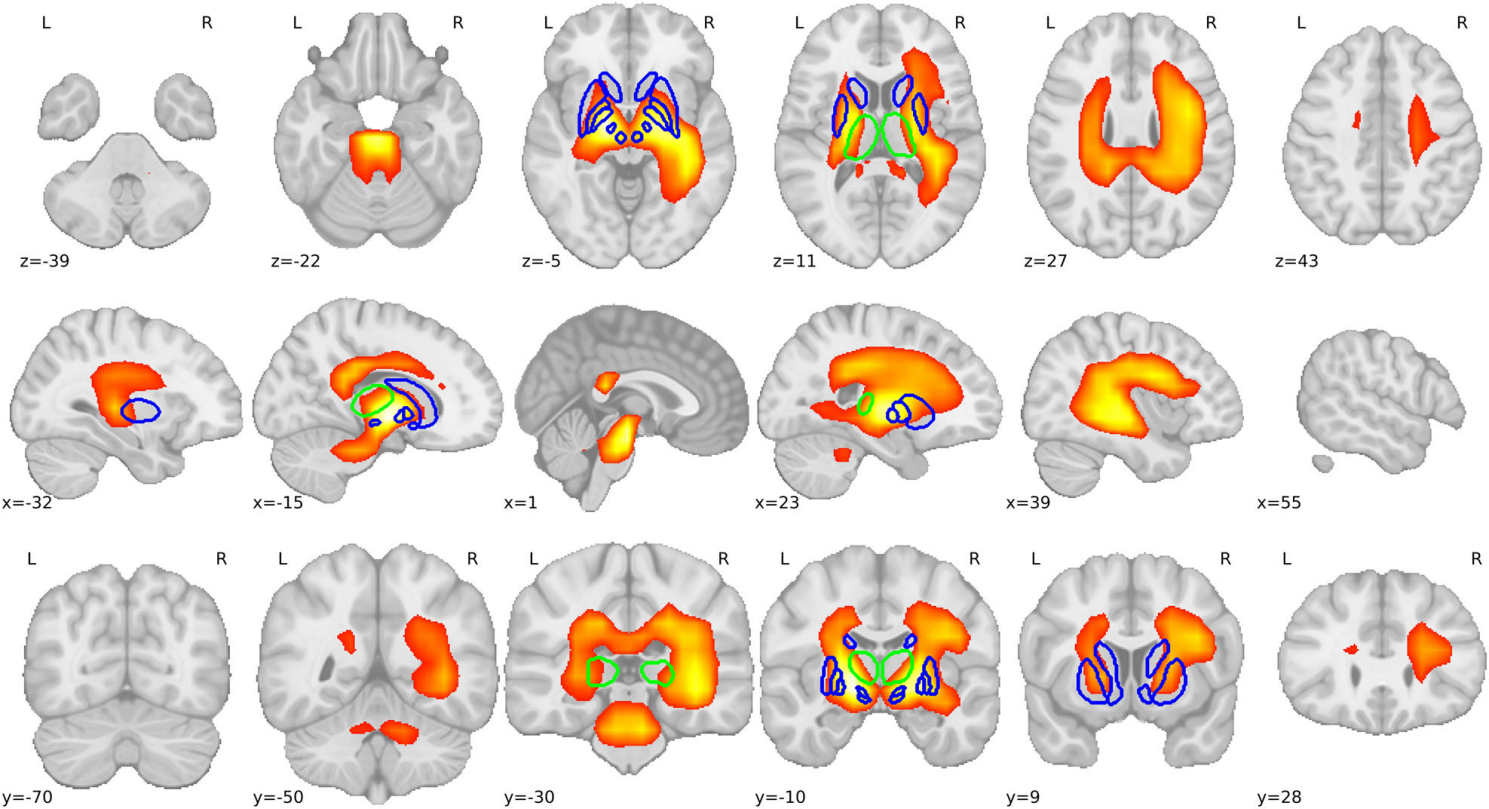
Activation map of MedicalNet extractor. The contours in blue represent predefined but dropped ROIs from the subcortical texture extraction method. The contours in green represent the thalamus, which was selected from subcortical texture features. The heatmap represents the cluster extracted by MedicalNet that survived the feature selection step, with an intensity threshold of 0.4

Using MedicalNet as a feature extractor, we visualized the surviving features after feature selection. The up-sampled saliency map from the last convolution layer revealed key ROIs that contributed to classification. The saliency map and most significant cluster are shown in Fig. 5. This dominant cluster identified several anatomical regions, including the superior temporal gyrus, cingulate gyrus, thalamus, putamen, GPe, GPi, hippocampus, insula, RN, SN, pons, and VTA.

## Discussion

In this project, we proposed a feature extraction method based on a pre-trained ResNet-based model. The features of this model outperformed previously published methods on both PPMI and external clinical datasets, demonstrating greater robustness and generalizability than clinical features. Our study also offers insights into the brain regions responsible for levodopa response prediction.

Multiple feature extraction methods were developed to maximize information extractable from T1-weighted MRI for LCT prediction. Although previous studies have demonstrated promising prediction performance using age-corrected regional gray matter intensity (accuracy 74%) and morphological graph (AUC 0.98) features, their conclusions raise uncertainties owing to the small sample sizes and lack of test and external validation sets [12, 13]. Subcortical ROI texture features (*r*^2^ of 0.76) employed clinical features alongside T1-weighted images with a relatively large sample size and an external validation set, although the imaging features were not evaluated separately [4]. Here, we developed a rigorous pipeline to re-evaluate previous methods with three feature combinations with or without both clinical and imaging features. Our results revealed that only the addition of subcortical texture features to the model would significantly improve the classification performance.

Although subcortical texture features showed predictive potential, we aimed to broaden our search for biomarkers beyond this region or with greater improvement. We modified MedicalNet to serve as a deep-learning feature extractor. The union model, incorporating MedicalNet-extracted features, outperformed all other methods across all three machine learning models on the test set (*p* < 0.001 for all). The saliency map, generated to visualize the selected features from MedicalNet, highlighted common subcortical ROIs (putamen, thalamus, GPi, GPe, RN, and SN) and additional ROIs (superior temporal gyrus, cingulate gyrus, hippocampus, insula, pons, and VTA). These findings potentially elucidate the superior performance of MedicalNet-extracted features over subcortical texture features. Gallagher et al [29] reported that subtle changes in anterior cingulate dopamine metabolism may contribute to dysexecutive behaviors in PD. Calabresi et al [30] proposed a link between the hippocampus and dopaminergic system changes in PD. Similarly, Faivre et al [31] suggested that VTA modulates motor and non-motor symptoms related to a partial loss of dopamine cells in PD. Halliday et al reported neuropathological changes in catecholamine cell groups in PD [32]. These findings suggest that the newly identified ROIs in our study may indeed be related to dopaminergic system changes in PD, explaining their contribution to LCT prediction. Although more related to cognitive impairment in PD, a D2 receptor loss was observed in the insula of PD patients, potentially affecting LCT results measured using MDS-UPDRS III [33]. For the superior temporal gyrus, no direct relationship with dopaminergic system changes in PD has been reported; however, its involvement with PD progression has been suggested [34].

Testing the external clinical set similarly, with only two feature sets previously established as predictive on the test set, a great decrease in performance was observed in the clinical and union models. This indicated potential bias in the clinical information of the external clinical set, which is common in the clinical environment. This drop in performance also questioned the generalizability of clinical information in real-world settings. However, the imaging model with MedicalNet outperformed all other models, with an AUC of 0.64 ± 0.03, demonstrating that the information extracted from objective T1-weighted MRI using MedicalNet was more robust and consistent than that of clinical information.

This study had some limitations. Although larger than that of several studies, our sample size was limited, which led to biased models that affected performance and eliminated the possibility of deep learning model training for specific tasks. Only one retrospective external clinical set limited the ability to further evaluate the generalizability of the predictive features. Although data-driven ROIs were identified via the MedicalNet extractor and validated using an external clinical set, their implication in levodopa response prediction and PD progression remains unclear, necessitating more interpretable models or features to elucidate their pathological roles. Lastly, considering the poor generalizability of clinical information, real-world prediction models need to rely on imaging features exclusively. However, using T1-weighted MRI alone yielded an AUC of 0.64 in the external clinical set, which implies the potential value of imaging data.

In conclusion, T1-weighted MRI offers more robust information than general demographic and clinical features. However, it may not suffice for predicting levodopa response in clinical settings (AUC 0.64 ± 0.03). Therefore, to improve practical LCT prediction performance, future studies should explore advanced imaging for robust feature extraction. A previous study highlighted the utility of T2* images in 16 subcortical ROI [4]. Subsequent studies could encompass an investigation of the predictive potential of our newly identified brain regions using T2* or quantitative susceptibility mapping which indicates the iron load [4], integrating this information to generate a more robust and generalizable model for levodopa response prediction.

### Supplementary Information

Electronic Supplementary Material

## Data Availability

The PPMI dataset is available at https://www.ppmi-info.org/. The external clinical dataset is available from the corresponding author upon reasonable request.

## Abbreviations

AUC: Area under the receiver operating characteristic curve
LCT: Levodopa challenge test
LEDD: Levodopa equivalent daily dose
PCA: Principal component analysis
PD: Parkinson’s disease
PPMI: Parkinson’s Progression Markers Initiative
ROIs: Regions of interest

## Code availability

The underlying code for this study is not publicly available but may be made available to qualified researchers on reasonable request from the corresponding author.
