# Supplementary material for: Unlocking the potential: T1-weighed MRI as a powerful predictor of levodopa response in Parkinson’s disease
Source: Insights Imaging. 2024 Jun 9;15:141. doi: 10.1186/s13244-024-01690-z (PMC11162980; doi:10.1186/s13244-024-01690-z)
Supplement: Supplementary file 1 — Electronic Supplementary Material [file 13244_2024_1690_MOESM1_ESM.pdf]

Unlocking the Potential: T1-Weighed MRI as a Powerful Predictor of Levodopa Response in Parkinson's Disease

ELECTRONIC SUPPLEMENTARY MATERIAL

Feature extraction

Previously published feature extraction methods are described in detail in the following three sections.

Age corrected regional gray matter intensity

Regional gray matter change, with age correction, was proven to be a potential biomarker for levodopa response prediction (1). We followed the steps of the previous study to perform age correction on CAT12 pipeline preprocessed images (2). For each voxel in healthy participants' gray matter, the intensity was regressed against age in GLMs to obtain a regression coefficient β:

$$y_H = X_H\beta + \varepsilon_H$$

where y_H is the intensity of a voxel in a healthy participant's gray matter, X_H is the age of the healthy participant, β is the regression coefficient, and ε_H is the residual. Therefore, for voxels in patients' gray matter, the age corrected intensity y_P^* was calculated as:

$$y_P^* = y_P - X_P\beta$$

The age correction was performed on both the training and test sets. Then a two-sample t-test was performed on each voxel to obtain the t-statistic map between good and bad responders in the training set, followed by an FDR correction with a *p*-value threshold of 0.05. The ROI of significant difference was treated as a mask to extract raw voxels from the age corrected gray matter images. To extract features robust to the noise, the first 50 principal components of the raw voxels calculated by PCA were used as features.

Subcortical ROI texture

The texture features, or the radiomic signatures of subcortical ROIs in T1 weighted MRI were reported to be not only predictive in PD diagnosis but also in LCT (3, 4). Here we used a PD25-subcortical-1mm atlas to extract 16 ROIs from the ANTs normalized T1 weighted images, including bilateral caudate, putamen, thalamus, GPi, GPe, STN, SN and RN. For

each ROI, the texture features were extracted by PyRadiomics (https://pyradiomics.readthedocs.io/en/latest/) with default settings. There were 86 texture features for each ROI, resulting in a total of 1,376 features for each sample.

Due to the possibility that these texture features were highly correlated, an additional step was performed to purge the highly correlated features. First, a correlation matrix of all features was calculated. Then feature pairs with an absolute correlation coefficient greater than 0.8 were identified. The feature having a smaller Spearman correlation coefficient with the LCT result in the identified feature pair was dropped. The remaining features were used for classification.

Morphologic brain network

The morphologic brain network, which was constructed by the morphologic similarity between brain regions, has been proven to be a potential biomarker for LCT prediction (5). We used CAT12 preprocessed gray matter images to construct the morphologic brain network. The cerebral cortex was first parcellated into 90 regions (45 per cerebellar hemisphere) based on AAL3 atlas. Then the PDF (probability density function) of voxel intensities of each ROI was calculated with KDE (kernel density estimation) method, after which Kullback–Leibler (KL) divergence was applied to build the morphologic brain network. The KL divergence between two ROIs, P and Q, was calculated as:

$$D_{KL}(P||Q) = \sum_i (P(i)log\frac{P(i)}{Q(i)} + Q(i)log\frac{Q(i)}{P(i)})$$

where $P(i)$ and $Q(i)$ are the PDF of ROI P and Q at voxel i respectively. The JS divergence was further calculated as:

$$D_{JS}(P||Q) = \frac{1}{2}D_{KL}(P||M) + \frac{1}{2}D_{KL}(Q||M)$$

where $M = \frac{1}{2}(P + Q)$. The morphologic brain network was constructed by the JS divergence between each pair of ROIs, which could be represented as a 90 by 90 adjacency matrix.

To generate the graph representation of the network, an additional sparsity threshold was applied to the adjacency matrix to filter out the weak connections. Two criteria were

determined to select the optimal threshold: (1) for each graph, its average degree should be greater than $2log(N)$, where N is the number of ROIs; (2) for each graph, the small-worldness metric σ should be greater than 1.1. If multiple subgraphs were generated after thresholding for each graph, the subgraph with the largest number of nodes would be selected and was recognized as the morphologic brain network of the sample. For a given network, several graph metrics were then calculated, including degree, degree centrality, betweenness centrality, nodal clustering coefficient, average clustering, global efficiency, local efficiency, characteristic path length, modularity score, sigma, nodal efficiency, and shortest path length.

References

1. Ballarini T, Mueller K, Albrecht F, et al. Regional Gray Matter Changes and Age Predict Individual Treatment Response in Parkinson's Disease. NeuroImage: Clinical 2019;21:101636.

2. Dukart J, Schroeter ML, Mueller K, and The Alzheimer's Disease Neuroimaging Initiative. Age Correction in Dementia – Matching to a Healthy Brain. PLoS ONE 2011;6. Ed. by Valdes-Sosa PA:e22193.

3. Chakraborty S, Aich S, and Kim HC. 3D Textural, Morphological and Statistical Analysis of Voxel of Interests in 3T MRI Scans for the Detection of Parkinson's Disease Using Artificial Neural Networks. Healthcare 2020;8:34.

4. the PREDISTIM Study Group, Betrouni N, Moreau C, et al. Can Dopamine Responsiveness Be Predicted in Parkinson's Disease Without an Acute Administration Test? Journal of Parkinson's Disease 2022;12:2179–90.

5. Xie Y, Gao C, Wu B, Peng L, Wu J, and Lang L. Morphologic Brain Network Predicts Levodopa Responsiveness in Parkinson Disease. Frontiers in Aging Neuroscience 2023;14:990913.
